# Proximate Composition, Physicochemical, and Lipids Profiling and Elemental Profiling of Rapeseed (*Brassica napus* L.) and Sunflower (*Helianthus annuus* L.) Grown in Morocco

**DOI:** 10.1155/2022/3505943

**Published:** 2022-09-09

**Authors:** Jamila Gagour, Moussa Nid Ahmed, Hasna Ait Bouzid, Samira Oubannin, Laila Bijla, Mohamed Ibourki, Ahmed Hajib, Jamal Koubachi, Hicham Harhar, Saïd Gharby

**Affiliations:** ^1^Biotechnology, Analytical Sciences, and Quality Control Team, Polydisciplinary Faculty of Taroudant, Ibn Zohr University, Taroudant 83000, Morocco; ^2^African Sustainable Agriculture Research Institute, Mohammed VI Polytechnic University, Laayoune, Morocco; ^3^Laboratory of Bioactive and Molecules of Interest National Agency of Medicinal and Aromatic Plants, Rabat, Morocco; ^4^Laboratory of Materials, Nanotechnology, and Environment, Faculty of Sciences, Mohammed V University, Av. Ibn Battouta, B.P1014, Rabat, Morocco

## Abstract

We investigate and compare the nutritional and physicochemical properties of rapeseed and sunflower grown in Morocco. In order to examine a complete physicochemical characterization, various parameters such as mineral profile, fatty acid composition, sterols contents, total flavonoids content (TFC), total polyphenols content (TPC), and quality oil parameters were evaluated. The results showed a relatively small difference in the physicochemical composition of the seeds, as sunflower seeds recorded higher amounts of protein and oil content (22.98 ± 0.01 g/100 g and 41.30 ± 0.50 g/100 g) than rapeseed (22.98 ± 0.01 and 38.80 ± 0.50), while mineral elements profile was observed to be statistically different. Nevertheless, both seeds were rich in K, Ca, P, Mg, and Na and they were relatively poor in Na, Fe, Mn, Cu, and Zn. The most represented macroelement was K with the amount of 7936.53 ± 63.87 mg/Kg in rapeseed and 7739.22 ± 59.50 mg/Kg in sunflower. On the other hand, Cu was present in the analyzed samples the least, mostly below 20 mg/kg. For TPC and TFC, the sunflower recorded higher values (49.73 ± 0.50 and 25.37 ± 0.39 mg GAE/g) than rapeseed (38.49 ± 0.24 and 22.55 ± 1.76 mg QE/g). The fatty acid composition showed that both extracted oils have beneficial proprieties, as they are rich in unsaturated fatty acids; namely, rapeseed oil contains a high level of oleic acid (C18 : 1) (62.19%), while sunflower oil was richer in linoleic acid (C18 : 2) (55.7%). As a result, we conclude that the studied varieties have major importance in terms of both nutritional and seed improvement potentials.

## 1. Introduction

Vegetable seeds and oils have served as the backbone of various agricultural economies throughout history [1]. Currently, oilseeds have long been in the spotlight [2], due to their patterns that reflect major trends in the modern world [2, 3]. Generally, oilseeds are the most important suppliers of high quality vegetable oils. Also, most of them provide significant nutritional value in the diet due to the high quality of proteins [3]; recognizing that both constituents are the main storage reserves of mature oilseeds, lipids are stored in the oil bodies and proteins in the protein bodies [4].

Vegetable oils and seeds are composed of a complex mixture of other compounds, which provided vital roles in the comfort of human life in various aspects [5], especially as sources of bioactive compounds such as natural antioxidants, vitamins, flavonoids, minerals, dietary fiber, and carbohydrates [6, 7]. When consumed in appropriate amounts, they can reduce the risk of cancer development, as they are associated with a wide range of health benefits, such as antimutagenic effect, anti-inflammatory properties, reduction of low-density lipoprotein synthesis, and antioxidant properties [6].

This market includes a wide variety of species, among which sunflower (*Helianthus annuus*) and rapeseed *(Brassica napus*) are considered as one of the most crucial oilseeds corps [8]. Oilseeds cover about 23% of the world's cultivated land [9]; the large area of cultivation of rapeseed and sunflower at the global level allows a very intersecting production rate reaching in the current campaign about 70 MT and 60 MT, respectively[10], ranking them as the second and third most productive oilseed crops worldwide, forming, together with soybeans, the most cultivated triangle in the world. In Morocco, sunflower remains an important annual oilseed grown in the country [11]. Also, rapeseed was cultivated for the first time in 1981 in Morocco [11]. The cultivation has shown a good suitability in different areas, mainly humid and subhumid, such as the Gharb region [12]. Currently, rapeseed is gaining importance as one of the main components of the oilseeds sector in Morocco. According to the latest available data, both oilseed crops (sunflower and rapeseed) are cultivated on an area of roughly 36,000 ha, with a total production of nearly 39,000 tons [13].

To the best of our knowledge, no information has been reported on the rapeseed and sunflower grown in Morocco; hence the originality of this research work appears. Therefore, here we seek to investigate the proximate composition, physicochemical, lipids profiling, and elemental profiling of both oilseeds.

## 2. Materials and Methods

### 2.1. Plant Material

This study was conducted using rapeseed and sunflower cultivars grown in Morocco. The mentioned cultivars were harvested in northwestern Morocco (the region of Rabat-Salé-Kenitra) in 2019. The samples were collected at the stage of full maturity; they were cleaned and sorted, and impurities such as strange particles (dust, sand, fibers....), as well as damaged seeds, were removed, before starting the different measurements.

### 2.2. Parameters Measured

#### 2.2.1. Mineral Elements (ICP-OES)

Dietary element content was measured using an ICP-OES spectrometer (Inductively Coupled Plasma Optical Emission Spectroscopy). The identification of the individual element content was reported based on absorptions measured at the following wavelengths: Ca content was detected at 422.7 nm, Mg at 285.2 nm, P at 666 nm, Na at 589.0 nm, K at 766.5 nm, Zn at 213.9 nm, Fe at 248.3 nm, Cu at 324.7 nm, and Mn at 279.5 nm. The results of the different elements were expressed in mg/kg (ppm) [14].

#### 2.2.2. Oil Content (OC)

The extraction of the oils was carried out using an appropriate apparatus of Soxhlet [15]. The powdered seeds (20 g) were extracted with pure n-hexane (200 mL) for 8 h. The solvent was partially removed in a vacuum rotary evaporator under reduced pressure. The oil was purged with nitrogen and stored at −4°C until further analysis and extraction yield (oil) was calculated gravimetrically.

#### 2.2.3. Moisture Content (MC)

Moisture content of each sample was measured by drying the weighed samples (about 2 g each), in an oven at 105°C ± 2°C, until a constant weight was reached. Following the methodology included in ISO 662 : 2016 standard, all the tests were performed in triplicate, and the means were reported [16].

#### 2.2.4. Ash Content (AC)

The ash content in the tested materials was performed as described by ISO 18122 : 2015. Briefly, 5 g of each sample was weighed into crucibles and then placed in a muffle furnace set at 500°C for 4 hours until whitish ash was obtained. The crucibles are weighed after cooling in a desiccator [17, 18].

#### 2.2.5. Protein Content (PC)

The proteins content was calculated by multiplying the total nitrogen content of the sample by a factor of 6.25 as described by ISO 4-1:1663, 2008. Indeed, the factor used to calculate the crude protein content from the total nitrogen content is derived from the Dumas method, which is the reference method for the determination of total nitrogen content [19].

#### 2.2.6. Total Carbohydrates Content (CC) and Energy Value (EV)

The carbohydrates were calculated by subtracting the difference between moisture, crude protein, ash, and fat from 100 percent according to Ibourki et al. [19].

Carbohydrate content (%) = 100−(Ash + Moisture +  Protein + Oil content).

Energy values were calculated from the data on total mineral content, protein, carbohydrates, and fats using factors of 4, 4, and 9 kcal/g, respectively [17].

EV (Kcal) = Proteins (g/100 g) × 4 + Carbohydrate (g/100 g) × 4 + Lipids (g/100 g) × 9.

#### 2.2.7. Extract Preparation

The extracts used to determine the total phenolic and flavonoids content and antioxidant activity were prepared by grinding 1 g of fresh sample and adding 10 ml of methanol 70%. The mixture was agitated on a shaker for 24 h at 20°C and filtered to be tested by different analyses.

#### 2.2.8. Total Polyphenol Content (TPC)

The total polyphenols content of seed polar extracts samples was measured by colorimetric assay following the protocol described by Chatoui *et al.* (2020) [18]. A mixture of 0.25 mL of extract solution was added to 1.25 mL of diluted Folin-Ciocalteu reagent (1 : 10, V/V). A 2.0 mL of sodium carbonate (Na_2_CO_3_: 7.5%) was added. The mixture was then incubated for 30 min in the water bath at 45°C. The absorbance was then measured at 765 nm by SCILOGEX SP-UV1100 spectrophotometer. All samples were prepared and measured twice, and the TPC was expressed as mg gallic acid equivalent/gram of dry matter (mg GAE/g DM).

#### 2.2.9. Total Flavonoid Content (TFC)

The total flavonoid content of the samples was measured by the colorimetric assay following the protocol described by Park et al. (2019) [20]. In a 10 mL test flask, 1 mL of the seed polar extracts solution was mixed with 0.3 mL of NaNO_2_ (0.5 N) and allowed to stand at room temperature for 5 minutes; thus, the mixture was added to 0.3 mL of AlCl_3_.6H_2_O (10%). Then, after 6 min, the mixture was added to 1 mL of NaOH (2N) and completed with distilled water up to the gauge line. The absorbance of the samples was measured at 415 nm using a SCILOGEX SP-UV1100 spectrophotometer. The TFC was expressed in terms of quercetin equivalent/g of dry matter (mg QE/g DM). This process was performed in duplicate for each extract.

#### 2.2.10. Antioxidant Activity (DPPH)

The antioxidant activity was determined using the 2,2-diphenyl-1-picrylhydrazyl (DPPH) method based on quantifying the free radical scavenging activity of the extracts described by Xu *et al.* (2016) [21], with some modifications. An ethanolic solution of DPPH (0.2 mM) was prepared and stored in an amber bottle to be protected from sunlight. Seed polar extracts were diluted 1 : 1000 (v/v) with ethanol. A 4 mL of diluted samples compound was added to 0.8 mL of DPPH solution. The whole mixture was then incubated in the dark for 30 minutes. The absorbance was measured versus a blank at 517 nm using a SCILOGEX SP-UV1100 spectrophotometer. Free radical inhibition by DPPH in percent was calculated using the following formula:

(1) $$\begin{array}{c} \text{Inhibition of }DPPH\%=\frac{A_{b}\_A_{a}}{A_{b}}*100, \end{array}$$

where *A_b_* stands for the absorbance of the negative control and *A_a_* stands for the absorbance of the sample. Results are average values ± standard deviations and are expressed as mg ascorbic acid equivalent/g DM.

#### 2.2.11. Trolox Equivalent Antioxidant Capacity (ABTS)

The ABTS of the samples was determined according to the method previously described by Ismaili *et al.* (2016) [22]. ABTS radical cation (ABTS^+•^) was produced by reacting ABTS solution dissolved in deionized water to a 2 mM concentration with 100 *μ*L of 70 mM potassium persulfate (K2S2O8). The solution was incubated in the dark at room temperature for 16 h before being used. The ABTS^+•^ solution was then diluted with ethanol until an absorbance of 0.70 at *λ* = 734 nm. Then, the samples were prepared by adding 200 *μ*L of extracts to 2 mL of the ABTS solution diluted with ethanol and allowed to react for 1 min. The absorbances were measured at 734 nm. The antioxidant activities of ABTS of the samples were expressed as mg of standard Trolox equivalent per g of dry matter (mg TE/g DM).

#### 2.2.12. Reducing Antioxidant Power (FRAP)

The reducing power of the samples that reflected their antioxidant activity was determined using the method previously described by Aryal *et al.* (2019) [23]. Briefly, 1 mL of the extract solution was added to 2.5 mL of 0.2 M sodium phosphate buffer (pH 6.6) and 2.5 mL of 1% (w/v) potassium ferricyanide (K_3_Fe (CN)_6_) solution. Thereafter, the mixture was incubated at 50°C for 20 minutes, followed by the addition of 2.5 mL 10% (w/v) trichloroacetic acid. Subsequently, the mixture was centrifuged at 3000 rpm for 10 minutes. The supernatant of the solution (2.5 mL) was mixed with deionized water (2.5 mL) and ferric chloride (FeCl_3_) (0.5 mL 0.1% w/v). Upon 30 min of incubation, the solution was measured at *λ*_max_ 700 nm against a blank. The reducing power of the extracts was expressed as mg of standard Trolox equivalent per g of dry matter (mg TE/g DM).

#### 2.2.13. Quality Oil Parameters

Physicochemical quality parameters, such as free fatty acid (acidity), peroxide value (PV), p-anisidine value (p-AnV), and UV specific extinction coefficients (K232 and K270) were determined according to the official analytical methods ISO 660:2009, ISO 3960:2017, ISO 6885:2006, and ISO 3656:201, respectively. Acidity and peroxide value (PV) were expressed in % oleic acid and milliequivalents of active oxygen per kilogram of oil (mEqO_2_/kg), respectively.

Extinction coefficients K232 and K270 were expressed as the specific extinctions of a 1% (w/v) solution of oil in cyclohexane measured in a 1 cm cuvette, using a SCILOGEX SP-UV1100 spectrometer. To measure the p-AnV, a solution of the oil in isooctane was reacted with p-anisidine standard in glacial acetic acid to form yellowish reaction products. The p-AnV was then analyzed from the absorbance measured at 350 nm, in a 1 cm cuvette, using a SCILOGEX SP-UV1100 spectrometer.

The total oxidation (Totox) value was calculated from peroxide and p-anisidine indexes using the formula: Totox = 2PV + p-AnV.

#### 2.2.14. Pigments Content

The pigments content of extracted oils samples was measured following the protocol described by Mazaheri *et al.* (2019) [24]. 7.5 g of each sample was accurately weighed, dissolved in cyclohexane, and taken to a final volume of 25 mL. Carotene and chlorophyll pigments were measured by determining the absorbance at 470 and 670 nm, respectively. A is the absorbance and *L* is the thickness of the spectrophotometer cell (10 mm) in the following equations:

(2) $$\begin{array}{c} \text{Chlorophyll}\left(\frac{mg}{100g}\right)\left(\frac{\left(A670*106\right)}{613}\right)*100L, \end{array}$$

(3) $$\begin{array}{c} \text{Carotenoid}\left(\frac{mg}{100g}\right)\left(\frac{\left(A470*106\right)}{2000}\right)*100L. \end{array}$$

#### 2.2.15. Fatty Acid Composition

The fatty acids were converted into corresponding fatty acid methyl esters (FAME) through transmethylation according to the standard (ISO 12966-2:2017). The method involved hydrolyzing esterified fatty acids to free fatty acids with methanolic sodium hydroxide (MeOH/NaOH) (2N). The composition of fatty acids was investigated in the form of their corresponding methyl esters by gas chromatography (Agilent 6890) coupled to a flame ionization detector (GC-FID). Capillary column CP-Wax 52CB (30 m × 250 *μ*m i.d., 0.25 *μ*m film thickness) was used. Helium (with a flow rate of 1 mL/min) was used as a carrier gas. The temperatures of the oven, injector, and detector were 185, 200, and 230°C, respectively. The injection volume of the samples was 1 µL in a split mode (split ratio 1 : 50) [19]. Results were expressed as the relative percentage of the area of each fatty acid peak.

The iodine value (IV) was computed from unsaturated fatty acids percentages using the following formula: IV = (% C16 : 1 × 1.001) + (% C18 : 1 × 0.899) + (%C18 : 2 × 1.814)+ (% C18 : 3 × 2.737) [15].

### 2.3. Phytosterol Composition

The composition of the phytosterol was measured according to ISO 12228-1:2014, following trimethylsilylation of the crude sterol fraction, using a Varian 3800 instrument equipped with a VF-1 ms (30 m, 0.25 mm i.d, 0.25 *μ*m film thickness), and using helium (flow rate 1.6 mL/min) as carrier gas. The temperature of the column was isothermal at 270°C; the temperature of the injector and detector was 300°C. Identification was based on retention time. Results were expressed as the relative percentage of the area of each phytosterol peak (g/100 g).

### 2.4. Statistical Analyses

The results presented are averages of analyses performed in duplicate or triplicate. These results are presented as mean ± standard deviation. The separation of mean values was done by the ANOVA test at the 0.05 level of significance.

## 3. Results and Discussion

### 3.1. Mineral Composition of Oilseeds

Oilseeds are a valuable source of mineral elements. In general, the intake of minerals in appropriate amounts is necessary for a vital life in humans [25]. Furthermore, the maintenance of dietary mineral homeostasis in the human body is very crucial, as they are involved in several mechanisms in basic biological processes [26].

Ten minerals, namely, Ca, P, Mg, K Na, Fe, Mn, Cu, Zn, and B, were analyzed during this study; the obtained results are summarized in Table 1.

As expected, according to the obtained results, the macroelements Ca, K, Mg, and P were found in high levels in the two types of seeds. K was the most abundant elements, followed by P in the two types of seeds with concentrations about 7936.53 ± 63.87 mg·kg^−1^ and 4713.95 ± 13.26 mg·kg^−1^ in rapeseed samples with 7739.22 ± 59.50 mg·kg^−1^ and 4471.13 ± 79.46 mg·kg-^1^ in sunflower seeds, respectively. The third position is occupied by Ca in rapeseed and by Mg in sunflower seeds with concentrations 3794.78 ± 4.08 and 2795.92 ± 20.74 mg·kg^−1^. Our results were higher than those found by Kolláthová *et al.* (2019) [27], for Ca, but lesser for K, Mg, and P in rapeseed seeds. In sunflower seeds, the values found in this study for Ca and K were higher (73.4 ± 4.6–163.6 ± 7.8 mg kg^−1^) than those reported by Kowalska *et al.* (2019) [28] and higher than those reported by Nadeem *et al.* (2010) [29], for Mg (73.4 ± 4.6–163.6 ± 7.8 mg·kg^−1^).

Regarding the microelements (Na, Fe, Mn, Cu, Zn, and B), both analyzed seeds contain low levels (all below 100 mg·kg^−1^). In sunflower seeds, the values found in this study for Na, Fe, and Mn were lower (446.7, 68.61 mg·kg^−1^ and 29 ± 2.26 mg·kg^−1^) than those reported by Mansouri *et al.* (2010) [29].

### 3.2. Physicochemical Characterization of Oilseeds

In addition to their richness of mineral elements, oilseeds are also characterized by a multitude of natural elements, which makes them a valuable source for consumers [30]. The ash, oil yield, water content, carbohydrate content, protein content, energy value, total polyphenol contents (TPC), and total flavonoid content (TFC) of both samples are shown in Table 2

#### 3.2.1. Ash Content

The ash content of rapeseed was found to be 3.61 ± 0.01 g/100 g, lower than the value found in sunflower (4.84 ± 0.02 g/100 g) (Table 2). Our finding results in sunflower were slightly higher (2.68–3.13 g/100 g) than those reported by Nadeem *et al.* (2010) [35] for Pakistani sunflower seeds and Mohammed *et al.* (2017) [36], for Chinese sunflower seeds (2.43 ± 0.09 g/100 g). However, the results of the ash content in rapeseed were similar (3.8 ± 0.25 g/100 g) to those reported by Alhomodi *et al.* (2021) [31], for rapeseed grown in France. The differences in crude ash content compared to literature sources may be due to topographical and climatic factors [27].

#### 3.2.2. Total Proteins Content

Proteins, regardless of their origin, all have the same role to play in the body. They have a structural role, as they are involved in most of our physiological processes [29]. These are localized in the cotyledons and embryonic axis of the seed, with only a small amount present in the integument [38].

According to the obtained results, the rapeseed and sunflower showed 20.8 ± 0.02 g/100 g and 22.98 ± 0.02 g/100 g of total proteins content, respectively. The high protein content of sunflower and rapeseed explains the recognition of these types of seeds as an excellent source of high quality seed-protein [32]. This makes them one of the most versatile crops in the world [39]. Our results were almost consistent with literature data [32, 36, 40], which revealed a protein content of 24.20 g/100 g, 24.64 g/100 g, and 24.2 g/100 g in sunflower seeds grown in Kingdom of Saudi Arabia and China, respectively. However, the proteins content in rapeseed was slightly lower than that in the results published by Alhomodi *et al.* (2021) and Radic *et al.* (2021) [31, 39], in rapeseed grown in the United States and Serbia, respectively.

#### 3.2.3. Moisture Content

Moisture content is an important factor in the stability of oilseeds, especially during storage [24]. This is also one of the interesting factors affecting the quality of the oil [41], as a high water content leads to the degradation of seeds, producing a rancid oil of poor quality [42]. The moisture content of rapeseed was 0.792 ± 0.048 g/100 g, significantly lower than the moisture content of sunflower (1.96 ± 0.03 g/100 g). The finding results in sunflower were widely lower (5.69 ± 0.11 g/100 g) than those reported by Mohammed *et al.* (2017) [36]. The difference in the moisture content between the samples and the literature may be due to variations in genetics, storage conditions, temperature, growing area, and several other factors between oilseeds [43].

#### 3.2.4. Oil Content

Oil content is one of the main criteria for assessing the effectiveness of oil production from oilseeds [44]. Knowing the oil yield of seeds is crucial to their value, as the monetary valuation in the economic oilseed trade is based on this value [45]. The yields of both samples (Table 2) were 38.8 ± 0.50 g/100 g (rapeseed) and 41.30 ± 0.50 g/100 g (sunflower). The results clearly indicated that both oilseeds could be considered as an excellent alternative source of oil. The finding results in sunflower seed were higher than the reported value of [36, 46], who revealed an oil yield of 37.47 g/100 g and 37.93 g/100 g, respectively. On the other hand, the present investigation of oil content was lower than the results previously published by Kirkegaard *et al.* (2018) [47], who reported an average oil content of 42.6–48.8 g/100 g in Australian oilseed rape, and Radic *et al.* (2021) [39], who reported a value of 45.96 g/100 g oil in the rapeseed grown in Bosnia and Herzegovina. These divergences in the literature for oil content can generally result from the various factors, such as climatic conditions, biological factor, environmental factor, soil, and crop management practices [24, 43].

#### 3.2.5. Energy Value and Carbohydrate Content

Carbohydrates, acting as stores in plants, appear as intermediates in biosynthetic pathways [48]. As shown in Table 2, the rapeseed and sunflower showed 31.42 ± 0.05 g/100 g and 33.45 ± 0.05 g/100 g of total carbohydrates, respectively (Table 2). Our finding results were lower to those reported by Mirpoor *et al.* (2021) [49], for rapeseed (38.5–41.3 g/100 g) and sunflower (44.8–51.7 g/100 g). However, the carbohydrates content recorded in sunflower was slightly higher (19.34 ± 1.22 g/100 g) compared to that reported by Salem *et al.* (2012) [40].

For energy value, the rapeseed and sunflower showed 580.44 ± 0.07 Kcal/100 g and 576.47 ± 0.15 Kcal/100 g, respectively. Our finding results were similar (572 Kcal/100 g) to those reported by Kotecka-Majchrzak *et al.* (2020) [32], for rapeseed and lower (620 Kcal/100 g) for sunflower. These differences in the nutritional value are generally explained by the difference in their protein, oil, and carbohydrates content [50].

#### 3.2.6. Total Phenolic Compounds (TPC) and Total Flavonoids Compounds (TFC)

The bioactive compounds such as phenols and flavonoids compounds present a diverse group of secondary metabolites that abundantly occur in the seeds kingdom. Phenolic compounds offer significant interest due to their antioxidant and anti-inflammatory properties [33]. Moreover, flavonoids are widespread seeds secondary metabolites, such as flavones, flavanols, and condensed tannins, which have various biological functions, such as antioxidant and free radical scavenging properties [51].

The obtained results showed a difference of the TPC between the examined samples (Table 2); sunflower recorded higher amount of TPC (49.73 ± 0.35 mg GAE/g DM), compared to rapeseed (38.49 ± 0.22 mg GAE/g DM). In contrast, the TFC values did not vary as much as the TPC, of which sunflower recorded a slightly higher content (25.37 ± 0.19 mg QE/g DM) compared to rapeseed (22.84 ± 0.46 mg QE/g DM). The values in the present study indicate that both seeds (rapeseed and sunflower) are rich in phenolic and flavonoids compounds. The authors Sladjana *et al.* (2010) [52] studied the total polyphenol content of three genotypes of Serbian sunflower cultivars. Indeed, they reported that the polyphenol content ranged from 14.68 to 18.24 mg GAE/g, which was lower than that found in this study. In addition, the amount of polyphenol reported in the present study was higher than the amount (5.1 mg GAE/g DM) reported by Tarasevičienė *et al.* (2019) [53]. Our finding results were also higher than those published previously [33, 51]. According to Faugno et al. [54], the TPC and TFC are highly dependent on the species, cultivar, climate, harvest time, and extraction method.

#### 3.2.7. DPPH Free Radical Scavenging Activity

The antioxidant properties of oils could be determined using a variety of approaches. Antioxidant compounds such as phenolics, flavonoids, tocopherols, and phytosterols that have strong antioxidant activity have their limitations for each radical [55]. In the present study, three complementary assays (DPPH, ABTS, and FRAP) were performed in order to have a comprehensive understanding of the antioxidant properties of the seed methanolic extracts of the samples, and the results are presented in Table 2.

The measurement by the DPPH method has been used extensively to quantify the antioxidant activity of polyphenols [56]. Antioxidant properties, especially radical scavenging activities, are extremely valuable owing to the essential role of free radicals in food and biological systems [57]. The antioxidant capacity DPPH of the samples expressed as the number of gram equivalents of ascorbic acid is shown in Table 2. The DPPH activity of the tested samples was 17.29 ± 1.23 mg AAE/g DM (rapeseed) and 4.61 ± 0.76 mg AAE/g DM (sunflower) (Table 2). Numerous studies have demonstrated that the antioxidant activity of seeds is strongly affected by the content of phenolic compounds, including TPC and TFC, and this relationship has been reported by other authors [58].

#### 3.2.8. ABTS and FRAP

ABTS is an additional method commonly used to determine the antioxidant potential. As shown in Table 2, sunflower recorded a higher value of ABTS (21.23 ± 0.25 mg·TE/g), compared to that recorded in 15.49 ± 0.21 mg·TE/g. The antioxidant activity of the samples seed extracts was also tested by the FRAP assay. The results showed that sunflower exhibited a higher value (11.98 ± 0.36 mg·TE/g) than rapeseed (7.73 ± 0.61 mg·TE/g). The authors in [59] worked on the antioxidant activity content of seven sunflower seed lines grown in Sudan and reported that the ABTS and FRAP values ranged from 19.46 to 30.70 mg·TE/g and from 29.65 ± 0.87 to 66.06 mg·TE/g, which were consistent with the values found in our study. However, the results of our study did not correlate with [60], who revealed average values of 15.1 and 26.9 mg·TE/g in ABTS and FRAP, respectively. According to Abdalla *et al.* (2021) [59], the antioxidant activity of sunflower seed would be assigned to the presence of the enzymatic antioxidants, phenolic compounds, carotenoids, l-ascorbic acid, and peptides, and, according to Mehmood et al. [61], the difference between the tested seeds occurred due to variation in the sample, variety, and experimental conditions.

### 3.3. Physicochemical Composition of Oilseed Oils

Quality parameters used regularly to measure the physical and chemical properties of extracted vegetable oils are content of free fatty acid (FFA), peroxide value (PV), P-anisidine value (p-AV), total oxidation (Totox), E232, E270 extinctions, pigment content, and iodine value as described in Table 3.

#### 3.3.1. Acidity

The rancidity of lipids is the main reason for introducing an unacceptable taste in oils. Consequently, it decreases the whole quality and shelf life of oils [62]. As these vegetable oils are sensitive to hydrolysis and oxidation, the question of the relevance of using free acidity as a marker of lipid-based degradation in vegetable oils is raised [63].

As shown in Table 3, the rapeseed recorded a higher amount of free fatty acids (1.41 ± 0.10 g/100 g), compared to sunflower (0.77 ± 0.07 g/100 g) (Table 3). The findings obtained were lower than those reported by Kreps *et al.* (2014) [66] for sunflower oil and higher than that found by Siger *et al.* (2015) [34] for rapeseed oil. High levels of free fatty acids (FFA) in oils can promote the oxidation [69]. According to Gotor and Rhazi (2016) [69], the levels of free fatty acids depend on the maturity and conditions of harvesting and storage of the oilseeds.

#### 3.3.2. Peroxide Value (PV)

Oxidation is the principal process that leads to the oxidative deterioration of oils and involves a free radical chain reaction with initiation, propagation, and termination stages [70]. To evaluate this process, several markers and methods are available: determination of the peroxide index, para-anisidine index, extinctions K232, K270, and the “Totox” (total oxidation value).

The peroxide value provides information on the concentration of hydroperoxide (the main products of primary oxidation) [70]. Thus, it represents a very practical criterion with a satisfactory sensitivity to assess the early stages of oxidative deterioration [71].

The relative initial values of peroxide index found in this study are almost similar (Table-3), and they were in the order of 3.70 ± 0.39 mEq O_2_/Kg for rapeseed and 3.65 ± 0.25 mEq O_2_/Kgfor sunflower oil, respectively.

In order to monitor the initial stages of oxidation, peroxide value (PV) is commonly applied to measure the total hydroperoxides content and to control the oxidation process [72]. In general, both values of samples were all below the limit recommended by the standards (10 mEq O_2_/Kg) [73]. Our results were almost comparable to those reported by Kreps *et al.* (2014) [64], for rapeseed oil. On the contrary, for sunflower oil, the finding results were lower than those reported by Kreps *et al.* (2014) and Mohammed *et al.* (2017), [36, 64].

#### 3.3.3. UV-Light Absorption (K232 and K270)

UV absorption at 232 nm (E232) and 270 nm (E270) is valuable rapid method for assessing the presence of oxidation products. Indeed, the verification of the peroxide value can be done by determining the UV absorbance at 232 nm, which is correlated to the presence of primary oxidation products [74]. On the other hand, the extinction E270 provides information on the degree of formation of secondary oxidation products. Thus, the specific extinctions at 232 nm and 270 nm of oil show clearly its oxidation state [72].

The analysis of specific extinctions showed a slight difference between samples. Within K232 and K270, the sunflower seemed to have lower values (2.22 ± 0.01 and 0.30 ± 0.01, respectively), while rapeseed recorded higher ones (2.55 ± 0.04 and 0.30 ± 0.01) (Table 3). The results obtained were in agreement with those found by Kreps et al. [64]. According to Gharby et al. [75], the more the value of extinction at 232 nm is important, the more the oil is peroxidized; similarly, the more the value of extinction at 270 nm is important, the more the oil is rich in secondary oxidation products.

#### 3.3.4. P-Anisidine Value (p-AnV)

As the hydroperoxide content decreases in the secondary phase of oxidation, a low peroxide value is not necessarily a quality indicator [76]. Therefore, it is important to combine this index with the measurement of the secondary products of oxidation to evaluate the actual oxidation status of oils. This is achieved by measuring the para-anisidine index, which detects the presence of nonvolatile aldehyde compounds generated during the decomposition of hydroperoxides [77].

The samples analyzed in this study recorded low p-AnV (Table 3), less than 1. The p-AV value for sunflower oil was 0.95 ± 0.01, higher than the p-AV recorded for rapeseed oil (0.77 ± 0.01). The finding results for rapeseed were similar to those reported by Wroniak *et al.* (2008) [65]. On the contrary, for sunflower, the finding results were lower than those reported by Mohammed *et al.* (2017) [36]. According to the literature, oil should have a p-AV index of less than 10 [78].

#### 3.3.5. Total Oxidation (Totox)

The overall level of oxidation of oils can be calculated by the Totox index, which represents an oxidative stability factor correlated to the peroxide index and the p-anisidine index [79]. In fact, the peroxide value is an indicator of the start of oxidation: it increases to reach a peak and then decreases when the oxidation state is advanced. The peroxides formed are transformed into volatile and nonvolatile aldehydic compounds which can be linked to another index, the anisidine index [80]. However, the Totox parameter, which is the sum of two times the peroxide value, and the anisidine value therefore give a precise idea of the oxidation state of fatty acids [80]. The total oxidative values of the analyzed oils were quite similar, and they were in the order of 8.11 ± 0.20 (sunflower oil) and 8.35 ± 0.10 (rapeseed oil) (Table 3). The obtained results for both samples were in line with the previous studies [65, 68]. In general, vegetable oils with a Totox value < 10 are considered fresh and of high quality [80].

#### 3.3.6. Pigments Content

The quality of oils can be assessed based on the content of specific substances such as chlorophylls and carotenoids that influence the stability of oils [81]. Indeed, pigments play an important role in the oxidative stability of oils due to their antioxidant activity; they prevent the autooxidation of vegetable oils [82]. The results of chlorophyll analysis were 0.03 ± 0.01 mg/100 g for sunflower and 0.47 ± 0.05 mg/100 g for rapeseed (Table 3). Although chlorophyll plays an important role in oxidative stability, according to Różańska et al. [83], it may lead to prooxidative effects in the presence of light. Carotenoids, unlike chlorophyll, are desirable in oils because of their antioxidant properties. Similarly, the *β*-carotene content was 0.57 ± 0.04 mg/100 g in rapeseed oil, higher than sunflower, which recorded 0.04 ± 0.01 mg/100 g. The obtained results were in line with the previous studies. Namely, Rękas *et al.* (2016) [84] recorded 0.66 to 1.7 mg/100 g in the carotenoids content which mainly composed of carotene and xanthophylls occurring in the cold pressed rapeseed oils from different varieties. Chew *et al.* 2020 [85], similarly reported a range of 2.94–35.8 mg/100 g in the carotenoids content from different studies.

#### 3.3.7. Iodine Value

The iodine value highlights the degree of oil's unsaturation (double bonds present in fats and oils) [86]. More specifically, the presence of the number of unsaturation in fatty acids established constituting vegetable oils.

As shown in Table 3, the iodine value of sunflower was 129.80 ± 0.03 g I_2_/100 g, significantly higher than that found in rapeseed oil (114.09 ± 0.10 g I_2_/100 g). These values were within the limits of the iodine index set by the standards [73]. Lužaić *et al.* (2021) [68] examined the IV of 25 sunflower oil samples and obtained a range of values starting from 81 ± 2.04 gI_2_/100 g to 127 ± 5.38  I_2_/100 g. The higher IV value of sunflower oil in the present study probably indicates a higher content of oleic fatty acid in its composition [68]. Consequently, the latter oils have interesting nutritional properties and pharmacological activity [15]. However, the oils with a high content of unsaturated fatty acids are more sensitive to oxidation [77].

#### 3.3.8. Fatty Acid Composition

Vegetable oils are characterized mainly by the fatty acid levels that they contain; these fatty acid composition reflects their stability [87], but, in the case of edible oils, it is also an essential indicator of their nutritional value [42]. The fatty acid profile showed the typical distribution of fatty acids in rapeseed and sunflower (Table 4). Interestingly, both oils stand out by their high content of unsaturated fatty acids. As it can be seen, oleic acid (a MUFA) is the main component of rapeseed oil (62.19%), followed by linoleic acid (C18 : 2) (18.58%). Almost the same finding is observed within the researches of [85]. By presenting the typical fatty acid composition in standards [73], the rapeseed oil used in this study belongs to the type of low erucic acid rapeseed oil (C22 : 1); in further way, it is classified as high oleic acid rapeseed oil.

The latter oil is recognized as one of the healthiest vegetable oils based on its unique fatty acid profile [85]. These essential fatty acids play key roles in human metabolism but cannot be synthetized by the organism, so they have to be incorporated from exogenous sources [92]. In this regard, sunflower was the richest in PUFAs [87]; the content of linoleic acid (18 : 2) (omega‐6) in sunflower oil could be increased to 55.7%, being the main fatty acid in this oil, while, in the latter, oleic acid (C18 : 1) constituted the second most notable amount (31.2%). Linolenic acid, a highly oxidizable molecule [41, 70], is also present in very low concentration in sunflower oil (usually less than 0.3%) (Table 4). The results obtained in sunflower oil were in agreement with those found by Mwakasege *et al.* (2021) [88]. This small content of linolenic acid can be used to detect the adulteration of sunflower oil with other vegetable oils rich in linolenic acids, such as rapeseed (up to of 14%). Both oils also contain two primary saturated fatty acids (SFA) 3.5–12% for rapeseed oil and 8–15% for sunflower oil, namely, palmitic acid and stearic acid [73]. Other fatty acids such as myristic acid (C14 : 0), palmitoleic acid (C16 : 1), arachidic acid (C20 : 0), and behenic acid (C22 : 0) were found only in relatively lower quantities (Table 4). The amount of different fatty acids in oils was widely variable and depended on multiple factors. For instance, agronomic characteristics have a great influence, including the variety of the plants, the cultivation system, and the environmental conditions of the production area and the time of harvest (which may be related to the state of ripening) [93].

#### 3.3.9. Phytosterols Composition

Phytosterols, phenolic and tocopherols compounds, represent a small part present in rapeseed and sunflower oils as in all vegetable oils [7] and constitute the majority of the unsaponifiable components of vegetable oils [2]. Although they represent only a minor part, they are known by their chemical characteristics and especially their antioxidant properties; they also bring a nutritional value and determine the organoleptic properties of edible oils. Also, these molecules can be used as an adulteration marker in certain oils such as argan oil [70] and olive oil [94]. Plant sterols possess several health benefits, such as lowering total blood cholesterol, leading to a significant reduction in cardiovascular disease risk [7].

Eight types of phytosterol compounds were detected in both samples. As shown in Table 4, *β*-sitosterol was the predominant sterol in both types of oils; sunflower oil possessed higher levels of *β*-sitosterol (57.51 ± 0.01 g/100 g), closely followed by rapeseed (54.9 ± 5.5 g/100 g). This phytosterol is also characteristic of many other vegetable oils [45]. These findings for rapeseed oil were similar to those reported by Jabeur *et al.* (2021) [90]. Sunflower also recorded higher levels of Δ7-stigmasterol and Δ7-avenasterol (16.44 ± 0.01–5.41 ± 0.01 mg/100 g), as compared to rapeseed, which recorded low levels (0.9 ± 0.2–0.3 ± 0.2 g/100 g). Brassicasterol was detected only in rapeseed oil at level of 7.5 ± 1.5 g/100 g, and stigmasterol was detected only in sunflower oil at level of 9.09 ± 0.01 g/100 g. These findings were in accordance with the results reported for rapeseed and sunflower cultivars grown in China [2, 95]. The data for phytosterol showed a higher difference, which may be due to the difference in genetic species [95]. According to the literature, the difference in phytosterol levels may also be related to differences in growing and storage conditions and analytical methods [95].

## 4. Conclusions

The present investigation indicates that the rapeseed and sunflower grown in Morocco represent an excellent source of mineral elements. They are also rich in protein, oil, and carbohydrates. The analysis of the fatty acid composition showed that both oils have many beneficial proprieties as they are rich in unsaturated fatty acids, especially rapeseed oil due to its high level of oleic acid (C18 : 1). Nevertheless, sunflower oil was richer in linoleic acid (C18 : 2). Furthermore, after comparing all the investigated parameters of the analyzed oilseeds with the literature, significant differences in physicochemical properties were found. These differences are expected, and they could be explained by several factors such as origin, quality technological process, nutritional advantages, and practical uses. Therefore, the Moroccan rapeseed and sunflower oilseeds could be considered a suitable ingredient in industrial uses.

## Figures and Tables

**Table 1:** Mineral composition of rapeseed and sunflower seeds (mg.kg^−1^).

	Rapeseed	Sunflower	Rapeseed	Sunflower
	Our study		[27]	[28]
Ca	3 794.78 ± 4.08^a^	1 613.03 ± 11.24^b^	3 560 ± 5.00	563.3–817.1
K	7 936.53 ± 63.87^a^	7 739.22 ± 59.50^a^	8 690 ± 10.00	6 622–7807
Mg	2 964.03 ± 28.78^a^	2 795.92 ± 20.74^b^	3 350 ± 10.00	2 457–3141
P	4 713.95 ± 13.26^a^	4 471.13 ± 79.46^a^	6 910 ± 8.00	—
Na	67.53 ± 0.47^a^	15.24 ± 2.10^b^	170 ± 0.01	39.9–47.0
Fe	30.93 ± 2.80^a^	38.61 ± 3.35^a^	38.30 ± 2.83	59.5–77.9
Mn	18.09 ± 0.21^a^	14.69 ± 0.13^b^	15.56 ± 0.07	42.9–57.1
Cu	1.25 ± 0.01^a^	19.74 ± 0.23^b^	21.25 ± 1.23	2.3–4.9
Zn	21.46 ± 0.07^a^	56.42 ± 0.61^b^	47.30 ± 0.20	28.2–36.6
B	9.38 ± 0.25^a^	14.96 ± 0.22^b^	—	—

Values are expressed as mean ± SD. Different letters represent significant differences (*P* < 0.05) between the cultivars.

**Table 2:** Physicochemical composition and antioxidant activity of the studied oleaginous plants.

	Rapeseed	Sunflower	Rapeseed	Sunflower
	Our study		[31–34]	[32, 35–37]
Ash content (g/100 g)	4.84 ± 0.02^a^	3.61 ± 0.01^b^	3.8	2.68–3.13
Protein content (g/100 g)	20.85 ± 0.01^a^	22.98 ± 0.02^b^	25.7	17.18–22.96
Moisture content (g/100 g)	1.96 ± 0.03^a^	0.79 ± 0.05^b^	6.0–6.4	3.06–6.32
Oil yield (g/100 g)	38.80 ± 0.50^a^	41.30 ± 0.50^b^	40.6	37.93
Carbohydrate content (g/100 g)	31.42 ± 0.05^a^	33.45 ± 0.05^b^	20	44.8–51.7
Energy value (Kcal/100 g)	559,60 ± 4,49^a^	592,41 ± 5,02^b^	572	620
TPC (mg GAE/g DM)	38.49 ± 0.22^a^	49.73 ± 0.35^b^	59.17	39.38–41.75
TFC (mg QE/g DM)	22.84 ± 0.46^a^	25.37 ± 0.19^a^	16.41	25–45
DPPH (mg AAE/g DM)	4.61 ± 0.76^a^	17.29 ± 1.23^b^	—	—
ABTS (mg TE/g DM)	15.49 ± 0.21^a^	21.23 ± 0.25^b^	—	—
FRAP (mg TE/g DM)	7.73 ± 0.61^a^	11.98 ± 0.36^b^	—	—

DPPH: radical scavenging activity. TFC: total flavonoid content. TPC: total phenolic content. TE: Trolox equivalent. Values are expressed as mean ± SD. Different letters represent significant differences (*P* < 0.05) between the seeds.

**Table 3:** Physicochemical parameters of rapeseed and sunflower oils.

	Rapeseed	Sunflower	Rapeseed	Sunflower
	Our study		[64, 65]	[66–68]
Acidity (g/100 g) (AV) or FFA	1.41 ± 0.10^a^	0.77 ± 0.07^b^	1.21	1.24
Peroxide value (mEq O_2_/Kg) (PV)	3.7 ± 0.39^a^	3.65 ± 0.25^a^	3.08	3.12
K232	2.555 ± 0.04^a^	2.22 ± 0.01^a^	1.32	2.20
K270	0.544 ± 0.021^a^	0.309 ± 0.008^b^	0.1	1.27
P-Anisidine value (p-AV)	0.95 ± 0.11^a^	0.81 ± 0.12^a^	0.65	2.17
Total oxidation (Totox)	8.35 ± 0.10^a^	8.11 ± 0.20^a^	6.87	7.09
Chlorophylls content (mg/100 g)	0.47 ± 0.05a	0.027 ± 0.008^b^	4.449	0,432
Carotenoids content (mg/100 g)	0.74 ± 0.04^a^	0.045 ± 0.01^b^	6.30	2.47
Iodine value (g (I_2_)/100 g oil)	114.33 ± 0,03^a^	129.80 ± 0.03^b^	112.6	127

Values are expressed as mean ± SD. Different letters represent significant differences (*p* < 0.05) between the seeds.

**Table 4:** Fatty acids composition and sterols composition of rapeseed and sunflower oils.

	Rapeseed	Sunflower	Rapeseed	Sunflower	Rapeseed	Sunflower
Fatty acids (g/100 g oil)	Our study		[85]	[88, 89]	Norm	
Palmitic acid (C16 : 0)	4.78 ± 0.10^a^	6.8 ± 0.78^a^	3.53–4.62	5.94	**2.5–7.0**	**5.0–7.6**
Stearic acid (C18 : 0)	2.58 ± 0.50^a^	4.58 ± 0.12^a^	1.52–2.02	2.71	**0.8–3.0**	**2.7–6.5**
Oleic acid (C18 : 1)	62.19 ± 0.24^a^	31.2 ± 0.09^b^	61.02–76.64	35.29	**51.0–70.0**	**14.0–39.4**
Linoleic acid (C18 : 2)	18.58 ± 0.12^a^	55.7 ± 0.81^b^	7.74–21.04	54.62	**15.0–30.0**	**48.3–74.0**
Linolenic acid (C18 : 3)	8.69 ± 0.09^a^	0.14 ± 0.04^b^	7.42–11.45	0.07	**5.0–14.0**	**ND-0.3**
Arachidic acid (C20 : 0)	0.82 ± 0.03^a^	0.10 ± 0.01^a^	0.48–0.64	0.20	**0.2–1.2**	**0.1–0.5**
Gadoleic acid (C20 : 1)	1.34 ± 0.01	ND	1.17–1.32	0.15	**0.1–4.3**	**ND-0.3**
SFA^*∗*^	8.18 ± 0.69^a^	11.44 ± 0.87^a^	5.54–7.03	11.0	**3.5–12**	**8–15**
MUFA^*∗∗*^	64.53 ± 0.25^a^	31.2 ± 0.09^b^	62.21–77.95	35.7	**51–76**	**14–39**
PUFA^*∗∗∗*^	27.22 ± 0.16^a^	55.84 ± 0.85^b^	15.12–30.91	53.0	**20–45**	**48–74**

Sterols (g/100 g oil)	Our study		[90]	[91]	Norm	

Cholesterol	0.3 ± 0.1^a^	0.16 ± 0.01^a^	0.38	ND	**ND-1.3**	**ND-0.7**
Brassicasterol	7.5 ± 1.5	ND	10.40	ND	**5.0–13.0**	**ND-0.2**
Campesterol	29 ± 2.5^a^	9.35 ± 0.01^b^	34.42	9.47–9.80	**24.7–38.6**	**6.5–13.0**
*β*-Sitosterol	54.9 ± 4.5^a^	57.51 ± 1.09^a^	53.98	59.66–64.66	**45.1–57.9**	**50–70**
Stigmasterol	ND	9.09 ± 0.01	0.37	6.93–11.06	**0.2–1.0**	**6.0–13.0**
Δ-5-Avenasterol	4.5 ± 1.01^a^	2.02 ± 0.01^a^	ND	1.84–2.83	**2.5–6.6**	**ND-6.9**
Δ-7-Stigmasterol	0.9 ± 0.2^a^	16.44 ± 0.11^b^	0.16	8.55–10.19	**ND-1.3**	**6.5–24.0**
Δ-7-Avenasterol	0.3 ± 0.2^a^	5.41 ± 0.6^b^	0.09	4.00–4.22	**ND-0.8**	**3.0–7.5**
Others	2.6	ND	ND	3.28–3.54	**ND-4.2**	**ND-5.3**

Data were recorded as mean values ± standard deviations. Different superscript letters within each column indicate a significant difference in each oil at the *p* < 0.05 level**. ND** represents not being detected. **SFA**^*∗*^**:** saturated fatty acids. **MUFA**^*∗∗*^**:** monounsaturated fatty acids**. PUFA**^*∗∗∗*^**:** polyunsaturated fatty acids.

## Data Availability

The data used to support the findings of this study are included within the article.
